# Insight of novel biomarkers for papillary thyroid carcinoma through multiomics

**DOI:** 10.3389/fonc.2023.1269751

**Published:** 2023-09-19

**Authors:** Wei Liu, Junkan Zhu, Zhen Wu, Yongxiang Yin, Qiao Wu, Yiming Wu, Jiaojiao Zheng, Cong Wang, Hongyan Chen, Talal Jamil Qazi, Jun Wu, Yuqing Zhang, Houbao Liu, Jingmin Yang, Daru Lu, Xumin Zhang, Zhilong Ai

**Affiliations:** ^1^ Department of Surgery (Thyroid & Breast), Zhongshan Hospital, Fudan University, Shanghai, China; ^2^ School of Basic Medical Sciences, Fudan University, Shanghai, China; ^3^ State Key Laboratory of Genetic Engineering, School of Life Sciences, Fudan University, Shanghai, China; ^4^ Department of Pathology, Wuxi Maternal and Child Health Care Hospital, Womens Hospital of Jiangnan University, Jiangnan University, Jiangsu, China; ^5^ Shanghai WeHealth BioMedical Technology Co., Ltd., Shanghai, China; ^6^ Department of General Surgery, Zhongshan Hospital (Xiamen), Fudan University, Xiamen, China; ^7^ Xiamen Clinical Research Center for Cancer Therapy, Xiamen, China; ^8^ Department of Biomedical Engineering, Balochistan University of Engineering and Technology, Khuzdar, Pakistan; ^9^ Chinese Academy of Sciences Center for Excellence in Molecular Cell Science, Cell Bank, Shanghai Institute of Biochemistry and Cell Biology, Chinese Academy of Sciences, University of Chinese Academy of Sciences, Shanghai, China; ^10^ Department of General Surgery, Zhongshan Hospital, Fudan University, Shanghai, China; ^11^ National Health Commission Key Laboratory of Birth Defects and Reproductive Health, Chongqing Population and Family Planning Science and Technology Research Institute, Chongqing, China

**Keywords:** papillary thyroid carcinoma, landscape, multiomics, biomarkers, prediction, diagnosis, prognosis

## Abstract

**Introduction:**

The overdiagnosing of papillary thyroid carcinoma (PTC) in China necessitates the development of an evidence-based diagnosis and prognosis strategy in line with precision medicine. A landscape of PTC in Chinese cohorts is needed to provide comprehensiveness.

**Methods:**

6 paired PTC samples were employed for whole-exome sequencing, RNA sequencing, and data-dependent acquisition mass spectrum analysis. Weighted gene co-expression network analysis and protein-protein interactions networks were used to screen for hub genes. Moreover, we verified the hub genes' diagnostic and prognostic potential using online databases. Logistic regression was employed to construct a diagnostic model, and we evaluated its efficacy and specificity based on TCGA-THCA and GEO datasets.

**Results:**

The basic multiomics landscape of PTC among local patients were drawn. The similarities and differences were compared between the Chinese cohort and TCGA-THCA cohorts, including the identification of PNPLA5 as a driver gene in addition to BRAF mutation. Besides, we found 572 differentially expressed genes and 79 differentially expressed proteins. Through integrative analysis, we identified 17 hub genes for prognosis and diagnosis of PTC. Four of these genes, ABR, AHNAK2, GPX1, and TPO, were used to construct a diagnostic model with high accuracy, explicitly targeting PTC (AUC=0.969/0.959 in training/test sets).

**Discussion:**

Multiomics analysis of the Chinese cohort demonstrated significant distinctions compared to TCGA-THCA cohorts, highlighting the unique genetic characteristics of Chinese individuals with PTC. The novel biomarkers, holding potential for diagnosis and prognosis of PTC, were identified. Furthermore, these biomarkers provide a valuable tool for precise medicine, especially for immunotherapeutic or nanomedicine based cancer therapy.

## 1 Introduction

Thyroid cancer (TC) is a prevalent malignancy within the endocrine system, with its subtypes classified based on histopathological patterns and derived cells (1). Amongst these subtypes, papillary thyroid carcinoma (PTC) is the most commonly diagnosed, particularly in China (37.7% estimated number of new cases in 2020, from http://gco.iarc.fr/), where it accounts for approximately 95.1% of all thyroid cancers (2, 3). Despite favorable postoperative outcomes, about 25% of PTC patients experienced a relapse during long-term follow-up, according to a retrospective study (4). Moreover, abuse of thyroid ultrasound and extensive examinations has led to overdiagnosing of PTC, underscoring the need for more evidence-based biomarkers for precise diagnosis and comprehensive prognostic prediction (5).

Traditionally, mutations in MAPK-related genes, including *BRAF* and *RAS* mutations, indicate the potential for dedifferentiation, aggressiveness, and angiogenesis of PTC (6, 7). Advance in high-resolution sequencing provides greater insights into the molecular profiles of PTC beyond *BRAF* mutations. In 2014, a comprehensive analysis, with 496 samples, of the genomic landscape of PTC was conducted, revealing a range of novel genetic alterations and oncogenic processes (8). Subsequently, various somatic and germline variations, including *CHEK2*, *NF1*, *ANK3*, *PMS2*, and even mtDNA point mutations, were predicted to drive disease-specific tumor development under different clinicopathological features using whole-exome sequencing (WES) (9–13).

RNA sequencing (RNA-seq) was employed to subclassify highly heterogeneous *BRAF*-mutated PTC, based on similar transcriptomic features, into clusters that were respectively associated with specific pathological patterns (14). Additionally, single-cell RNA sequencing (scRNA-seq) was used to explore the landscape of the PTC tumor environment (15). In 2022, Guo et al. used 1724 FFPE (Formalin Fixed Paraffin Embedded) samples for diagnostic of thyroid cancer by integrating high-throughput proteomics with protein biomarkers (16).The combination of omics data has enabled promising genotype-phenotype crosstalks.

Despite the potential of multiomics approaches for screening PTC metastasis and stratification, current biomarkers for recurrence and precise diagnosis remain scanty. This study applied WES, RNA-seq, and data-dependent acquisition (DDA) to screen for corresponding molecular targets in PTC. 17 genes were identified as biomarkers for predicting the prognosis and diagnosing PTC. Among these genes, *ABR*, *AHNAK2*, *GPX1*, and *TPO* were specifically utilized to construct a diagnostic prediction model. This model demonstrated distinct specificity and efficacy, enabling the accurate differentiation between tumor and non-tumor samples which was expected to be a valuable tool for diagnosing of PTC. In conclusion, in order to identify novel biomarkers for prognosis and diagnosis in local patients, we preliminarily investigated the molecular properties of PTC through 6 matched samples from Chinese patients.

## 2 Materials and methods

The detailed materials and methods are attached in **Supplementary Material**.

## 3 Results

### 3.1 The landscape of somatic alterations of papillary thyroid carcinoma among local patients

**Figure 1:**
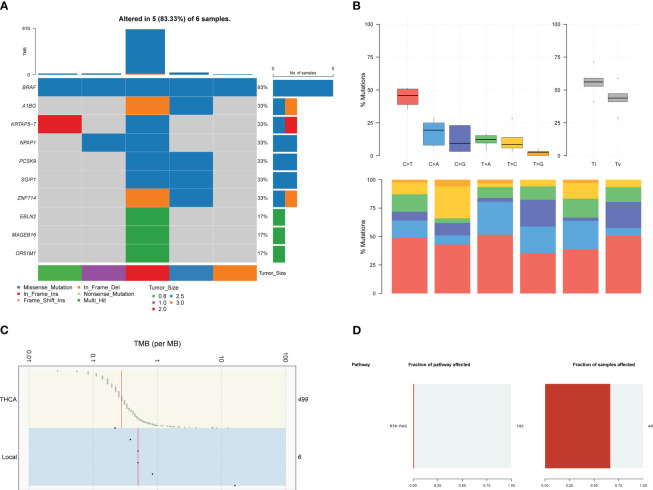
The results of WES in local patients. **(A)**. The genomic profiles of local patients by waterfall plot. Top: mutation counts of the top 10 mutated genes in each patient. Media: the top 10 mutated genes and their occurrence in 5 patients. Bottom: the tumor size of each patients, mutation types, and the mutation types frequencies are demonstrated by a bar plot in the right panel. **(B)** The frequency of types of somatic mutations, missense mutation, SNP, and C > T were the main SNV types. **(C)** Tumor mutation burden of local patients and TCGA-THCA cohorts. **(D)** Pathway associated with driver genes in somatic mutations, the RTK-RAS pathway was affected in 4 of six samples.

Germline alterations were filtered out from the tumor samples using the Genome Analysis Toolkit (GATK), focusing on retaining somatic alterations. WES revealed that our local patients’ dominant variant classification, variant type, and single nucleotide variant (SNV) class were consistent with the profiles observed in the TCGA-THCA (Thyroid Carcinoma, THCA)cohorts (**Figure S2**). However, there were notable differences in the top mutated genes. While canonical *BRAF* mutations were observed in over 60% of our patients, other top mutated genes in our cases included *OR51M1*, *MAGEB16*, *EBLN2*, *ZNF714*, *SGIP1*, *PCSK9*, *NPAP1*, *KRTAP5-7*, and *A1BG* (**Figures 1A**, **S2A**), instead of *NRAS* and *HRAS* observed in TCGA-THCA (**Figure S2B**). The frequency of different types of mutations was analyzed, revealing a higher frequency of transitions compared to transversions (**Figure 1B**).

Furthermore, genomic data indicated a higher tumor mutation burden (TMB) in our local patients compared to the TCGA-THCA dataset (**Figure 1C**). Subsequently, we examined the driver mutations in our local patients. *BRAF* (Z-score=5.546, p<0.001) and *PNPLA5* (Z-score=5.546, p<0.001), a gene closely associated with lipid metabolism (17), were the only two genes implicated in tumor pathogenesis (**Table 1**). Regarding pathways related to tumor activities, only the RTK-RAS pathway was significantly affected due to *BRAF* mutations (**Figure 1D**). However, it is essential to note that our sequencing technique focused on the exonic regions and may have provided limited insights into gene fusions and chromosomal aberrations occurring in intronic or other non-exonic regions. Further investigation using complementary techniques, such as whole genome sequencing or Long-read-sequencing analysis, may be necessary to better understand these genomic alterations in PTC.

**Table 1:** Characteristics of driver genes of the somatic mutations in local patients*.

Hugo Symbol	Mutated Samples	Z-score	Pvalue	FDR	Fract_muts
BRAF	4	5.546	<0.001	<0.001	1
PNPLA5	1	5.546	<0.001	<0.001	1

^*^: Cancer driver genes were identified through R package maftools by oncodriveCLUST algorithm, the concept of which is based on the fact that most of the variants in cancer causing genes have a tendency to be enriched at hotspot loci (18, 19).

### 3.2 Gene expression landscape of normal thyroid tissue and papillary thyroid carcinoma

**Figure 2:**
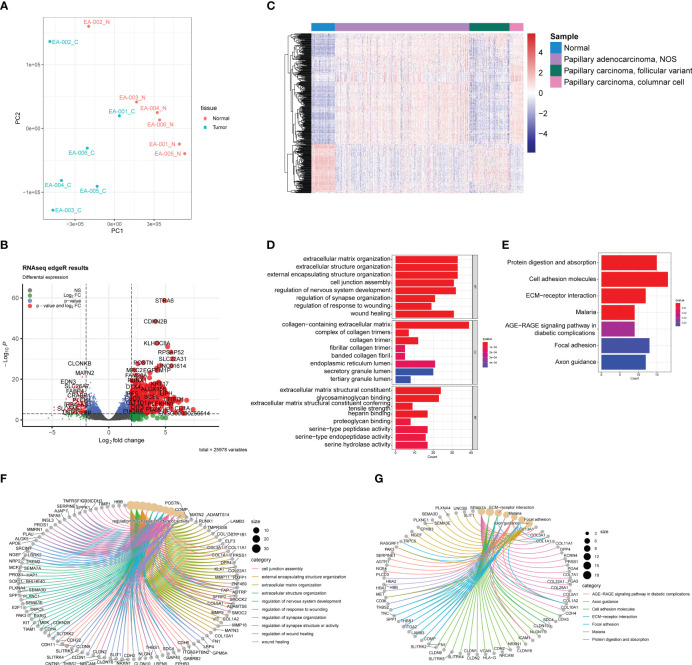
Transcriptomic analysis based on RNA-seq between normal samples and PTC. **(A)** PCA plot of normal samples (red) and PTC (blue). **(B)** Volcano plot exhibits 572 DEGs (353 upregulated and 219 downreglated) with red plots. **(C)** Heatmap of DEGs based on TCGA-THCA cohorts. The pathway enrichment analysis were achieved through **(D)** GO analysis and **(E)** KEGG analysis. **(F)** The gene-concept networks visualized the results of GO analysis, and of **(G)** KEGG analysis.

A total of 25,978 non-low expression genes’ transcriptomic profile was analyzed using an R script after filtering out genes with transcript per million mapped (RPKM) values less than 3. Principal component analysis (PCA), using the FactoMineR R package, revealed a clear distinction in gene expression between normal and tumor samples (**Figure 2A**). Except for one paired sample (EA-002), the normal samples exhibited a convergence compared to the tumor samples, while tumor samples showed relative heterogeneity. The log2 fold changes and corresponding false discovery rates (FDR) to identify differentially expressed genes (DEGs), employing criteria of |log2 (fold change) | > 2 between normal and tumor samples and FDR < 0.001. 572 unique genes were identified, using the edgeR R package, as DEGs, consisting of 353 upregulated genes and 219 downregulated genes, as illustrated in the volcano plot (**Figure 2B**).

To validate the ability of the identified DEGs to accurately reflect the gene expression differences between normal tissue and tumors, we selected samples from the TCGA-THCA dataset. Specifically, based on their clinical information, we included 59 normal samples, 339 papillary adenocarcinoma-NOS (not otherwise specified), 101 papillary carcinoma-follicular variant, and 35 papillary carcinoma-columnar cell samples (**Figure 2C**). The heatmap demonstrated that the DEGs we identified successfully distinguished normal samples from most tumor samples (**Figure 2C**). However, it is noteworthy that the transcriptomic profiles of normal samples and follicular variants of papillary thyroid carcinoma (FVPTC) appeared similar in the heatmap. This observation may be attributed to the low risk of adverse outcomes associated with FVPTC (20).

We performed Gene Ontology (GO) and Kyoto Encyclopedia of Genes and Genomes (KEGG) enrichment analyses to explore the potential mechanisms underlying PTC (**Figures 2D, E**). The GO analysis identified the top eight significant pathways in biological processes, cellular components, and molecular functions. Notably, these pathways were predominantly related to the extracellular matrix (ECM), including extracellular matrix organization, collagen-containing extracellular matrix, and extracellular matrix structural constituent (**Figure 2D**). Previous studies have highlighted the dysregulation of ECM in PTC, which is closely associated with tumor activities such as migration and invasion (21). In addition, the KEGG analysis revealed seven significant pathways, including protein digestion and absorption, cell adhesion molecules, ECM-receptor interaction, malaria, AGE-RAGE signaling pathway in diabetic complications, focal adhesion, and axon guidance. Of particular interest is the interaction between ECM and cell adhesion (**Figure 2E**), the latter of which has been reported in several studies to play a crucial role in tumor growth and invasion (22, 23). Furthermore, it is intriguing that the GO and KEGG analyses unveiled some neuronal system-related pathways, potentially indicating the presence of neuronal cell-adhesion molecules in PTC, such as *NrCAM* (24). The gene-concept network helps illustrate how specific genes are linked to various pathways and concepts (**Figures 2F, G**), highlighting their potential roles in PTC development and progression.

### 3.3 Weighted gene co-expression network analysis

**Figure 3:**
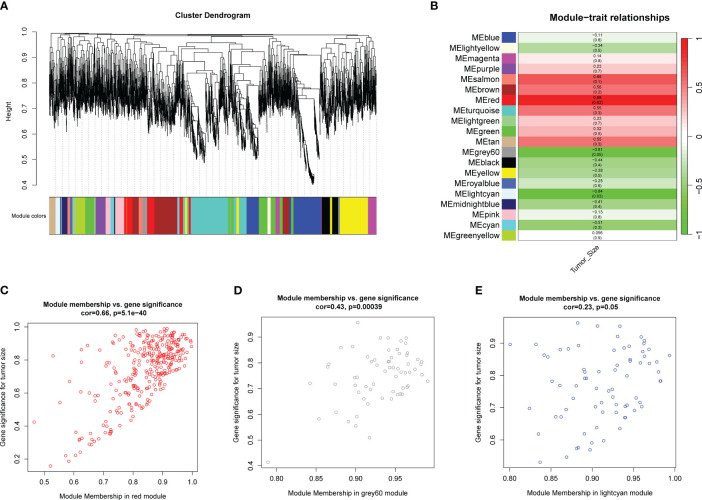
WGCNA analysis using genes with FDR<0.05 in differential analysis. **(A)** Cluster dendrogram shows modules derived from genes’ expression characteristics. **(B)** Module-trait relationships demonstrate the correlation between modules and tumor size. **(C)** MM vs GG scatter plots of MEred (r=0.66, p<0.001), **(D)** MEgrey60 (r=0.43, p<0.001), and **(E)** MElightcyan (r=0.23, p=0.05).

To identify potential hub genes for further validation, a loose screening criterion was applied for DEGs, selecting genes with an FDR < 0.05 in the differential analysis. A total of 4,828 genes from the 6 tumor samples were then used to construct a co-expression network. The analysis was performed using the R package WGCNA, where a soft-threshold power of 16 was chosen to ensure appropriate scale independence and mean connectivity of the network. Based on the resulting cluster dendrogram, genes with similar co-expression patterns were grouped into 20 modules (**Figure 3A**). Eigengenes were correlated with the clinical data to assess the correlation between modules and clinical characteristics (**Table 1**). Notably, the MEred module exhibited a high correlation with tumor size (r=0.89, p=0.02), while MEgrey60 (r=-0.81, p=0.05) and MElightcyan (r=-0.84, p=0.03) showed negative correlations (**Figure 3B**). Scatter plots were generated to depict the correlations between module membership (MM) and gene significance (GS) for the MEred (r=0.66, p<0.001), MEgrey60 (r=0.43, p<0.001), and MElightcyan (r=0.23, p=0.05) modules (**Figures 3C–E**), confirming the significant correlation of these modules with tumor size. Finally, we exported the edge data of genes within these three modules and utilized Cytoscape v3.10.0 for further analysis and visualization. This enabled a more detailed examination of the interactions and relationships among the genes within these modules.

### 3.4 The proteomic variance between normal tissue and papillary thyroid carcinoma

DDA was utilized for quantitative proteomic analysis. Before analysis, proteins with undetectable abundance or those without annotations in the NCBI Reference Sequence Database were excluded from the dataset. A total of 3,624 proteins were identified for further analysis.

**Figure 4:**
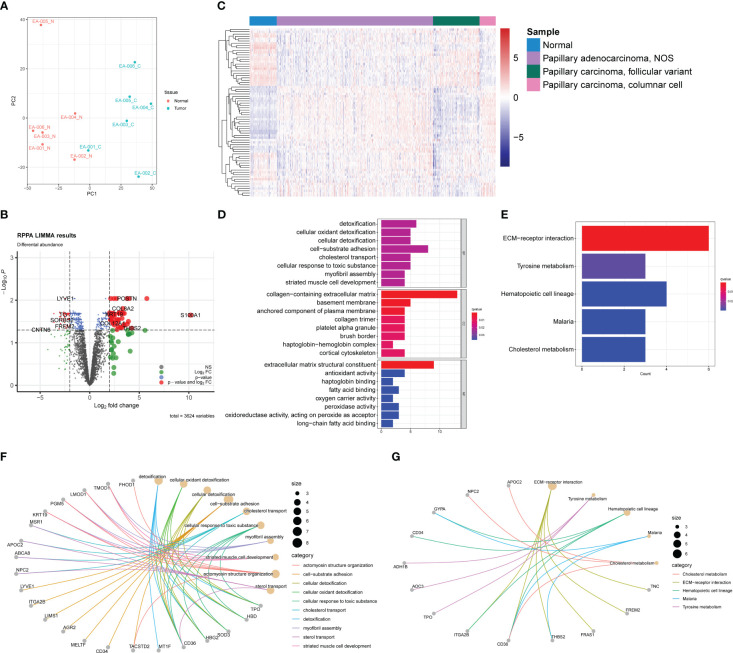
Proteomic profiles based on DDA between normal samples and PTC. **(A)** PCA plot of normal samples (red) and PTC (blue). **(B)** Volcano plot exhibits 79 DEPs (35 upregulated and 44 downregulated) with red plots. **(C)** Heatmap of DEPs based on TCGA-THCA cohorts. The pathway enrichment analysis were achieved through **(D)** GO analysis and **(E)** KEGG analysis. **(F)** The gene-concept networks visualized the results of GO analysis of and **(G)** KEGG analysis.

The PCA plot of the proteomic data demonstrated a distinct separation between normal and tumor samples, except sample EA-001_C, which appeared to be closer to the normal sample cluster (**Figure 4A**). This heterogeneity among the tumor samples was consistent with the findings in the transcriptomic PCA plot, indicating potential molecular variations within the PTC samples at the protein level.

To identify differentially expressed proteins (DEPs), we employed a similar strategy as used for DEGs. DEPs were defined using a threshold of |log2 (fold change) | > 2 and an acceptable FDR cutoff of < 0.05. Using the limma R package, we identified 79 DEPs, with 35 proteins upregulated and 44 proteins downregulated, as shown in the volcano plot (**Figure 4B**). Similar to the transcriptomic analysis, we extracted gene expression data corresponding to the DEPs from the TCGA-THCA dataset. As anticipated, the DEPs also demonstrated the capability to distinguish tumor samples from normal samples, although not as efficiently as the DEGs (**Figure 4C**). This observation could be attributed to the fact that direct protein abundance data were not utilized, which may have affected the discriminatory power of the DEPs. GO and KEGG enrichment analyses were conducted to investigate the functional implications of the DEPs further. Besides enriching ECM-related pathways, such as collagen-containing extracellular matrix, we observed frequent enrichment of detoxification-associated pathways, like cellular oxidant detoxification and lipid metabolism, including fatty acid binding and long-chain fatty acid binding (**Figure 4D**). KEGG analysis revealed five pathways in which the DEPs may participate: ECM-receptor interaction, tyrosine metabolism, hematopoietic cell lineage, malaria, and cholesterol metabolism (**Figure 4E**). These findings were partly consistent with the results from the GO analysis, suggesting potential roles of ECM, lipid metabolism, and detoxification processes in PTC. The gene-concept networks were also exhibited to visualize the link between proteins and specific pathways (**Figures 4F, G**).

### 3.5 Screening for hub genes for prognosis and diagnosis

**Figure 5:**
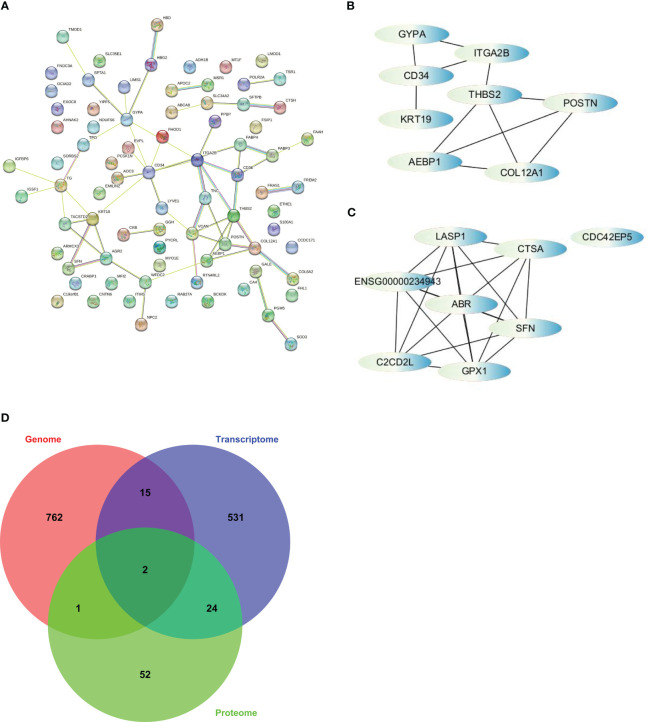
The strategy to screen for hub genes was based on cytoHubba, a plugin of Cytoscape. **(A)** PPI analysis finished on STRING. **(B)** Interactions of top 8 hub genes from PPI. **(C)** Interactions of top 8 hub genes from WGCNA. **(D)** Venn diagram illustrates overlap among somatic alterations, DEGs, and DEPs and revealed 2 hub genes based on integration of multi-omics profiles.

PPI analysis was performed by using the STRING database to explore protein-protein interactions (PPI) and identify potential hub genes. The resulting PPI networks were visualized (**Figure 5A**), and the edge file was downloaded and imported into Cytoscape. Similarly, we imported the edge file obtained from the previous WGCNA into Cytoscape to search for hub genes within the network.

To identify the hub genes, we utilized the cytoHubba app in Cytoscape and ranked the nodes based on maximal clique centrality (MCC), a measure of network connectivity. From the PPI analysis, we selected *AEBP1*, *CD34*, *COL12A1*, *GYPA*, *ITGA2B*, *KRT19*, *POSTN*, and *THBS2* as hub genes (**Figure 5B**). From the WGCNA analysis, we selected *ABR*, *C2CD2L*, *CDC42EP5*, *CTSA*, *GPX1*, *LASP1*, and *SFN* as hub genes (**Figure 5C**). However, *ENSG00000234943*, a lncRNA, was excluded from further analysis.

We examined the overlap among somatic alterations, DEGs, and DEPs to integrate the results from multiple omics analyses. Through this analysis, we identified two additional hub genes, *TPO* and *AHNAK2*, which had somatic alterations and were also differentially expressed at the transcriptional and protein levels (**Figure 5D**). Therefore, we obtained a set of 17 hub genes through the multi-omics analysis. These hub genes represent key network elements and merit further investigation to elucidate their functions in predicting the prognosis and diagnosis of PTC. Among the identified hub genes, *AEBP1*, *CD34*, *COL12A1*, *ITGA2B*, *THBS2*, and *TPO* exhibit lower expression levels in thyroid tumors. On the other hand, *AHNAK2*, *GPX1*, *KRT19*, and *SFN* show higher expression levels in thyroid tumors (**Figure S3**). These differential expression patterns suggest their potential as diagnostic markers for distinguishing thyroid tumors from normal tissue.

### 3.6 Potency validation of hub genes to predict prognosis and diagnosis of PTC

**Figure 6:**
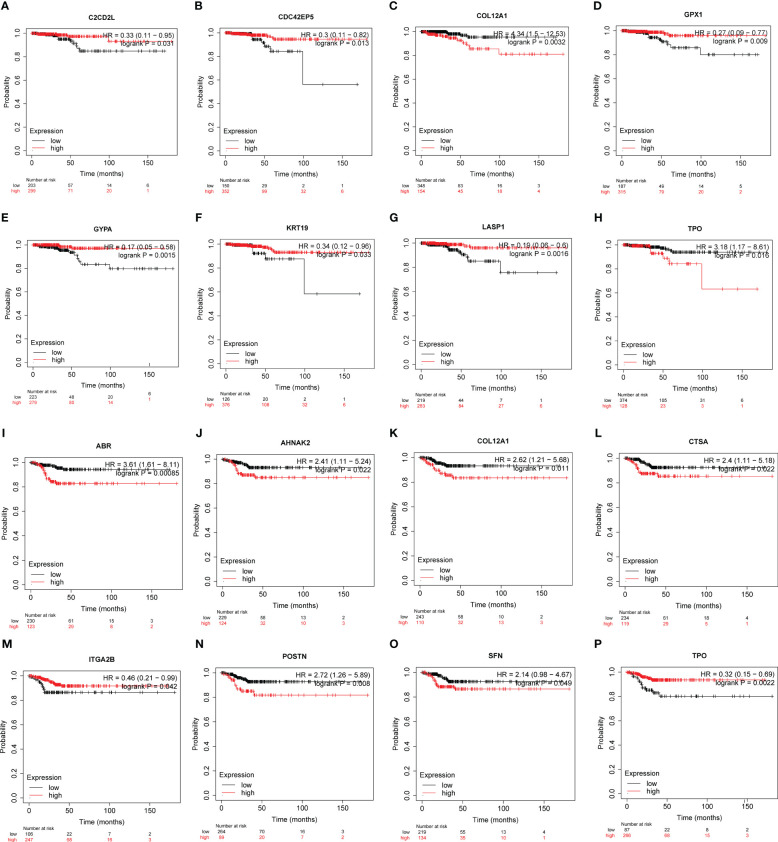
The potency of hub genes in predicting prognosis of PTC patients. Survival analysis on the K-M plotter based on 502 patients from TCGA-THCA cohorts revealed associations between the expression of hub genes and OS **(A-H)** or RFS **(I-P)**. **(A)** *C2CD2L*. **(B)** *CDC42EP5*. **(C)** *COL12A1*. **(D)** *GPX1*. **(E)** *GYPA*. **(F)** *KRT19*. **(G)** *LASP1*. **(H)** *TPO*. **(I)** *ABR*. **(J)** *AHNAK2*. **(K)** *COL12A1*. **(L)** *CTSA*. **(M)** *ITGA2B*. **(N)** *POSTN*. **(O)** *SFN*. and **(P)** *TPO*. Hub genes with significantly improved OS or RFS were shown.

Based on the Kaplan-Meier plotter employing TCGA-THCA (n=502), the associations between the expression of hub genes and patient survival were found (**Figures 6**, **S4**, **S5**):

Overall Survival (OS) (**Figures 6A–H**, **S4**):

- Low expression of *C2CD2L*, *CDC42EP5*, *GPX1*, *GYPA*, *KRT19*, *LASP1* was associated with better prognosis.
- High expression of *COL12A1* and *TPO* was associated with a better prognosis.

Recurrence-Free Survival (RFS) (**Figures 6I–P**, **S5**):

- High expression of *ABR*, *AHNAK2*, *COL12A1*, *CTSA*, *ITGA2B*, *TPO* was associated with better prognosis.
- Low *ITGA2B* and *TPO* expression was associated with a better prognosis.

These findings suggest that the expression levels of these hub genes may have predictive value in determining patient survival outcomes in papillary thyroid carcinoma.

**Figure 7:**
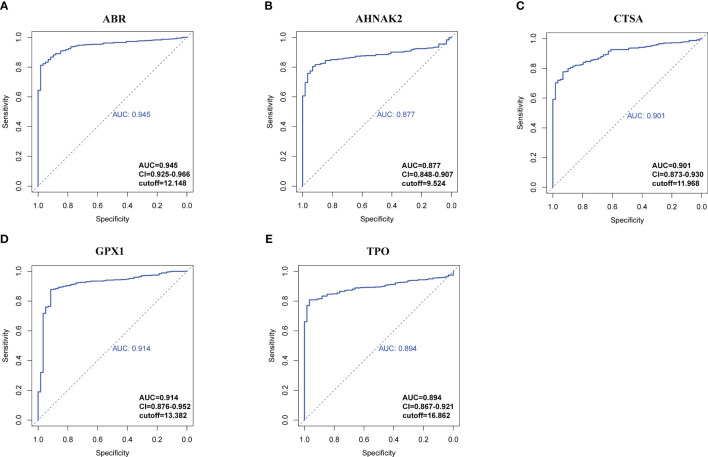
ROC analysis of hub genes with AUC (value > 0.85). **(A)** *ABR*. **(B)** *AHNAK2*. **(C)** *CTSA*. **(D)** *GPX1*. **(E)** *TPO*. Confidential intervals and respective cut-off values were also shown at the right-bottom of the picture.

RNA-seq data from 534 samples (475 PTC samples and 59 normal samples) obtained from the TCGA-THCA cohorts were analyzed to evaluate the diagnostic potential of the hub genes in PTC. First, we examined whether the expression levels of individual genes could distinguish between tumor and normal tissue. Using receiver operating characteristic curve (ROC) analysis, we assessed the performance of each gene in differentiating PTC from normal tissue to select genes qualified to be employed to construct a clinical predictive model (**Figures 7**, **S6**). According to the events per variable (EPV) rule, we considered the area under the curve (AUC) values above 0.85, suggesting these genes show promising diagnostic efficacy in controlling the number of variables. Five genes, *ABR* (AUC=0.945), *AHNAK2* (AUC=0.877), *CTSA* (AUC=0.901), *GPX1* (AUC=0.914), *TPO* (AUC=0.894), performed significantly in the ROC analysis (**Figure 7**). The high AUC values obtained from the ROC analysis support the potential utility of these genes in constructing a robust diagnostic model for PTC.

**Figure 8:**
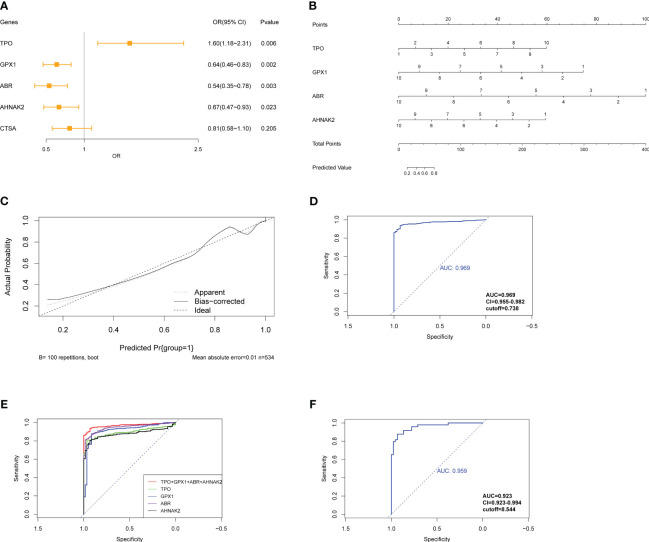
Construction of logistic regression model for diagnosis of PTC. **(A)** Forest plot exhibits OR and Pvalue of candidate genes for the diagnostic model. **(B)** Nomogram shows the association between genes’ expression and diagnosis of PTC. **(C)** The calibration curve shows good prediction potential of the model. **(D)** ROC analysis of model applied on TCGA-THCA cohorts and **(E)** compared with individual genes composed of the model. **(F)** ROC analysis of model applied on GSE33630.

To develop a diagnostic model for PTC, logistic regression analysis was performed using the glmnet R package. The gene expression data from the TCGA-THCA cohort was preprocessed by assigning scores of 1-10 based on percentile ranking within each gene’s expression data, with higher expression levels receiving lower scores. Tumor samples were given a score of 1, while normal samples were given 0. The logistic regression model was built using the expression levels of the following genes: *TPO*, *GPX1*, *ABR*, *AHNAK2*, and *CTSA*. The glmnet package with the “family=‘binomial’” parameter was used for logistic regression analysis. The odds ratio (OR) with its 95% confidence interval (95% CI) and p-values were calculated and presented in a forest plot (**Figure 8A**). Stepwise regression was performed using the step function. Among the genes, *ABR*, *AHNAK2*, *GPX1*, and *TPO* significantly associated with PTC diagnosis (p<0.05) and were identified as pivotal elements in the clinical model. Then, we achieved the formula of “y=0.4211*TPO-0.5256*GPX1-0.7043*ABR-0.4178*AHNAK2 + 13.8133”. A nomogram was constructed using the rms R package, incorporating these four genes as a reference for diagnosing PTC (**Figure 8B**). The calibration curve demonstrated good prediction efficacy of the model (**Figure 8C**). The diagnostic performance of the model was evaluated using ROC analysis, yielding an AUC value of 0.969 (**Figure 8D**), which outperformed the individual genes’ diagnostic potential (**Figures 7**, **8E**). External validation was conducted using the GSE33630 dataset (49 PTC and 45 normal samples), yielding an AUC value of 0.959 (**Figure 8F**), indicating the model’s capability to diagnose PTC.

Pan-cancer expression data of the four genes were exhibited for a further selection of other tumors to assess the model’s specificity for PTC (**Figure S7**). The model was applied to different types of tumors from the GSE63514 (cervical squamous cell carcinoma), GSE132305 (cholangiocarcinoma tumor), GSE53757 (kidney renal clear cell carcinoma), GSE121248 (liver hepatocellular carcinoma), and GSE43458 (lung adenocarcinoma) datasets. The model exhibited inferior diagnostic potential in these tumor types compared to PTC, confirming its specificity for PTC diagnosis (**Figure S8**). These results suggest that the diagnostic model based on the expression levels of *ABR*, *AHNAK2*, *GPX1*, and *TPO* has strong diagnostic efficacy for PTC and demonstrates specificity for PTC compared to other tumors.

## 4 Discussion

Fine needle aspiration biopsy (FNAB) is currently the most widely used diagnostic method for thyroid tumors, followed by cytologic examination and mutation detection to confirm the specific subtype (25). Common molecular characteristics such as *RET/PTC* rearrangement, *RAS*, and *BRAF* mutations have been identified as indications of PTC (26). Recent data-oriented analysis has further subclassified PTC into immune-enriched, *BRAF*-enriched, stromal, and CNV-enriched subtypes, providing valuable insights into precise medicine for PTC in China (14). However, most molecular biomarkers for PTC prognosis and diagnosis have been based on profiles from Western cohorts, lacking sufficient representation for the Chinese population based on a multi-omics landscape. This study observed distinct genomic characteristics in local patients compared to TCGA-THCA cohorts. In addition to the highly mutated *BRAF*, *PNPLA5* was identified as a somatic driver gene. Proteomic data revealed the potential impact of this lipid metabolism-associated gene on cholesterol binding at the protein level (27). Differential analysis of DEGs and DEPs allowed to distinguish between benign samples and PTC, and these findings were validated using TCGA-THCA cohorts. Furthermore, the research highlighted the heterogeneity of gene expression among different variants of PTC, emphasizing the need for further investigation into the molecular distinctions of PTC variants.

Subsequent functional enrichment analysis revealed pathways potentially involved in tumorigenesis and other tumor-related activities. For instance, previous studies have reported the significant role of ECM in promoting tumor growth and invasion by influencing gene expressions such as *uPAR* and *CREB3L1* (28, 29). Cell adhesion, a crucial process for tumor invasion, is closely associated with ECM degradation. Dysregulation of cell adhesion-associated molecules has been identified in the development of PTC: CD44 has been implicated in promoting tumor metastasis and lymphatic invasion (30), while *galectin-3* exhibits lower expression correlating with increased cancer metastasis potential but higher expression aiding PTC diagnosis (31). Previous omics research has also highlighted the enrichment of genes related to cell adhesion in PTC, emphasizing its pivotal role in tumor development (22, 32). Furthermore, disruptions in lipid metabolism may contribute to PTC progression. Integrated analyses involving lipidomics, proteomics, and metabolomics have demonstrated enhanced lipid metabolism reprogramming within PTC samples (33). Abnormal expression levels of specific molecules like *LPL*, *FATP2*, and *CPT1A* have been linked to tumor progression and poor prognosis (33). Our study also uncovered the enrichment of detoxification-associated pathways at the protein level. In PTC specifically, *NOX4* has been attributed to reactive oxygen species (ROS) production (34), while overexpression of *PIM-1* is thought to promote an antioxidant response that maintains an oxidant state conducive for tumors (35). However, further investigation is needed to fully understand how these pathways impact PTC activities.

Our study utilized a combination of WGCNA, PPI analysis, and integration of multi-omics profiles to identify 17 hub genes. These hub genes were further investigated for their potential to predict the diagnosis and prognosis of PTC. Interestingly, these hub genes were found to be primarily associated with pathways related to cell adhesion and ECM, which aligns with the enrichment analysis we previously conducted. Significant efforts have been dedicated to developing predictive models for central cervical lymph node metastasis (36–38) and prognosis based on radiomics, molecular characteristics, and other clinical baseline data (39–41). With ongoing advancements in FNAB techniques and ultrasonography, the rate of overdiagnosis of thyroid cancer reached over 80% in urban areas in China (5). Consequently, there is an urgent need for evidence-based and reliable approaches to diagnose PTC to evade the overuse of techniques accurately. Lu et al. utilized a metabolomics method for the diagnosis of papillary thyroid microcarcinoma, with AUC=0.992 model in local patients (42). Previously, Guo et al. exploited a protein-based neural network classifier for thyroid nodules, with AUC=0.93 in the training cohort and AUC=0.89 in the test cohort, encouraging improvement of cytopathology for PTC (16). Besides Chen et al. revealed a 3-gene panel for diagnosis of PTC through scRNA-seq based on TCGA-THCA cohorts and testified the efficacy of the diagnostic model in his own cohorts, exhibiting an AUC=1 (43). In our research, four genes (*ABR*, *AHNAK2*, *GPX1*, and *TPO*) were selected through multivariate logistic regression analysis to construct a diagnostic model. TPO is a crucial protein in thyroxine production that is nearly absent in thyroid cancers. It has been reported to indicate lymph node metastasis and recurrence in PTC (44). *AHNAK2* functions in cell adhesion and cell junction processes; it has also emerged as a novel prognostic factor for PTC and gastric cancer (45, 46). GPX1 belongs to the glutathione peroxidase family and is critical in maintaining redox balance. Altered expression of *GPX1* has been associated with tumorigenesis by regulating ROS levels that promote tumor survival (47). Our study discovered its prognostic and diagnostic value, specifically within PTC. Although *ABR* has received limited attention in cancer research, the results showed it highly expressed in PTC compared to normal samples, suggesting its potential diagnostic and prognostic value. Consequently, our model demonstrated excellent performance in diagnosing PTC with AUC=0.969 in TCGA-THCA cohorts, AUC=0.959 in test sets, and notable specificity for this type of tumor rather than others.

Although the screening of hub genes was conducted using local patient data, we mainly validated their diagnostic and prognostic potential on Western cohorts. Further research is necessary to validate the model, specifically on large Chinese populations. Additionally, due to limitations in sample size, our profiles were not comprehensive enough to capture the complete molecular characteristics of local PTC cases. To address this limitation and gain a more detailed understanding of the initiation and progression mechanisms of PTC, scRNA-seq and proteomic technology have been increasingly employed. ScRNA-seq approach allows for individual cell-level analysis and helps eliminate deviations caused by the mixed tumor and normal cells (15). Besides, scRNA-seq facilitates in-depth comprehension of the molecular characteristics and heterogeneity of PTC along with perspectives from the tumor microenvironment, cellular interactions, etc. This not only fosters more precise biomarker mining but also advances the accuracy of subclassification of patients. Moreover, it is important to note that our current model relies on categorized gene expression. Proteomic analysis was used in detected, quantified and qualitied proteins in tissue or serum samples of patients (48–53). High throughput mass spectrometry for proteomic, for example DIA, makes the leap from research to clinical application (16, 54, 55). To generalize these application, further work is required to develop a risk scoring system. Additionally, we also need a prospective cohort to validate the model.

## 5 Conclusions

In conclusion, our study successfully integrated genomic, transcriptomic, and proteomic landscapes to identify 17 hub genes with promising diagnostic and prognostic potential based on TCGA-THCA datasets. We have derived a specific predictive model for PTC composed of *ABR*, *AHNAK2*, *GPX1*, and *TPO* with expectations for future clinical diagnosis applications. These novel biomarkers may be the targets for immunotherapeutic or nanomedicine based cancer therapy. However, future research should focus on validating these findings in large Chinese populations while exploring more comprehensive molecular characterization approaches such as scRNA-seq analysis, which may provide insights into tumor immune infiltration and other tumor environment factors that could impact the heterogeneity of PTC. In addition, classical indexed like the level of thyroid hormones and the results of imaging results should be considered to construct the clinical model as well. Researchers should discuss the results and their interpretation regarding the previous studies and the working hypotheses. The findings and their implications should be addressed in the possible broadest context.

## Data availability statement

The datasets presented in this study can be found in online repositories. The names of the repository/repositories and accession number(s) can be found in the article/**Supplementary Material**.

## Ethics statement

The studies involving humans were approved by the ethical review board of the Zhongshan Hospital, Fudan University. The studies were conducted in accordance with the local legislation and institutional requirements. The participants provided their written informed consent to participate in this study.

## Author contributions

QW: Conceptualization, Data curation, Formal Analysis, Investigation, Methodology, Project administration, Writing – original draft, Writing – review & editing. WL: Data curation, Formal Analysis, Writing – original draft. JZ: Methodology, Software, Writing – original draft, Writing – review & editing. ZW: Formal Analysis, Funding acquisition, Methodology, Writing – original draft, Writing – review & editing. YY: Funding acquisition, Methodology, Validation, Writing – review & editing. YW: Methodology, Software, Writing – review & editing. JJZ: Data curation, Investigation, Visualization, Writing – review & editing. CW: Data curation, Funding acquisition, Visualization, Investigation, Writing – review & editing. HC: Data curation, Visualization, Writing – original draft. TQ: Writing – review & editing. JW: Data curation, Visualization, Methodology, Writing – review & editing. YZ: Data curation, Methodology, Writing – review & editing. HL: Supervision, Validation, Writing – original draft. JY: Resources, Supervision, Validation, Writing – original draft. DL: Funding acquisition, Supervision, Visualization, Writing – review & editing. XZ: Supervision, Writing – review & editing. ZA: Conceptualization, Funding acquisition, Project administration, Resources, Supervision, Validation, Visualization, Writing – review & editing.
