# Supplementary material for: Insight of novel biomarkers for papillary thyroid carcinoma through multiomics
Source: Front Oncol. 2023 Sep 19;13:1269751. doi: 10.3389/fonc.2023.1269751 (PMC10546062; doi:10.3389/fonc.2023.1269751)
Supplement: Supplementary file 1 [file DataSheet_1.pdf]

Supplementary Material

Contents

1 Materials 2
1.1 Patient samples of thyroid carcinoma 2
2 Methods 2
2.1 Samples preparation 2
2.2 Histopathology 2
2.3 Whole-exome sequencing 2
2.3.1 DNA extraction 2
2.3.2 Alignment 3
2.3.3 Variant detection and annotations 3
2.3.4 Calling significantly mutated genes 3
2.4 RNA-Seq 3
2.4.1 RNA extraction 3
2.4.2 RNA-Seq data analysis 4
2.5 Proteomic analysis 4
2.5.1 Protein extraction and FASP digestion 4
2.5.2 Nano-LC-MS/MS analysis 4
2.5.3 Database searching 5
2.6 Differential genes and protein analysis 5
2.7 Pathway enrichment analysis 5
2.8 Weighted gene co-expression network analysis(WGCNA) 5
2.9 Protein-protein interactions (PPI) network and screening for hub genes 6
2.10 Prognostic verification of hub genes 6
2.11 ROC analysis and logistic regression model 6
3 The raw data repository 6
4 Abbreviated terms 7
5 Reference 8
6 Supplementary Figures and Tables 10
6.1 Supplementary Figures 10
6.2 Supplementary Tables 17

1 Materials

1.1 Patient samples of thyroid carcinoma

The thyroid carcinoma samples used for this study were collected from Zhongshan Hospital in Shanghai, China. A single site of the primary tumor and paired peritumor tissues were collected. Patients were randomly selected upon their first visit and underwent no anticancer treatments before surgery. Primary tumor tissues and paired peritumor tissues (>0.5 cm apart from tumor edge) were surgically resected and transferred to liquid nitrogen. 6 paired specimens were collected with the clinical information, including gender, age, tumor size, degree of differentiation, lymph node metastasis, TNM staging, and so on (Tabal S1 clinical baseline information). The samples was randomly selected with the only criteria, which the primary tumor size must can be enough to completion of muitiomics research (the diameter >0.5 cm). All patient samples were obtained with the hospital's approval of the Research Ethics Committee, with written informed consent provided by all participants.

2 Methods

2.1 Samples preparation

A single site of the primary tumor and paired peritumor tissues were collected. Collected specimens were divided into four parts: the two parts were snap frozen in liquid nitrogen and then stored in a -80°C refrigerator until being used for DNA extraction and proteomics; the third part was processed with RNAlater Stabilization Solution (Invitrogen, catalog No: AM7021, Carlsbad, CA) and stored at -80°C until being used for the RNA extraction; the fourth part was treated as freezing microtome section for HE staining and used for histological evaluation.

2.2 Histopathology

Freezing microtome section specimens were HE-stained, examined and evaluated independently by experienced pathologists and information regarding tumor degree of differentiation, TNM staging, and tumor purity were provided. Normal samples represented thyroid tissue without abnormalities, while tumor samples represented authentic PTC tumor tissue, with at least 60% of the tissue comprising tumor cells. This rigorous selection process guarantees the authenticity and reliability of the subsequent analysis results, Figure S1.

2.3 Whole-exome sequencing

2.3.1 DNA extraction

Total DNA from Thyroid cancer tissues and paired peritumor tissues were extracted using the CretBiotech DNA extraction Kit (CretBiotech, Suzhou, China) according to the manufacturer's instructions. DNA degradation and contamination were monitored on 0.8% agarose gels. DNA concentration was measured using Qubit DNA Assay Kit in Qubit 2.0 Fluorometer (Life Technologies,CA, USA). A total amount of 0.5 mg genomic DNA per sample was used as input for DNA sequencing. Sequencing libraries were generated using the Agilent SureSelect Human All Exon V6 kit (Agilent Technologies, CA, USA) following the manufacturer's recommendations and barcodes were added to each sample. DNA fragments were sequenced on an Illumina HiSeq X ten sequencing system.

2.3.2 Alignment

The exome sequencing data were used to identify somatic variations found in tumor samples but not in paired peritumor tissues. The Trim_Galore script (version 0.4.4) was employed to perform adaptor trimming, and low-quality reads filtering. Clean reads were aligned via BWA MEM (version 0.7.12-r1039) against the human reference genome hs37d5 (based on GRCh37assembly with human virus sequences) with default parameters(1). Primitive alignment results were sorted and indexed through Samtools (version 1.5); they were then marked, and duplicate reads were removed by running picard (version 22)(2). We ran GATK (version 3.7) to perform local realignment and recalibration following the tool's best practice, and the output list was prepared for downstream analysis.

2.3.3 Variant detection and annotations

We used Mutect2 in GATK3.7, a variant caller, to detect possible SNVs and small indels (insertion/deletion) in the tumor genome. Mutect2 was run in the default setting by taking tumor and paired peritumor tissues as input and only mutations called in the targeted area were evaluated. Human SNP database (dbSNPv132) and COSMICv81 coding and noncoding mutation data were also provided as reference inputs of Mutect2. Passed mutations were annotated by the somatic mutation annotation software Oncotator (version 1.9.2.0).

2.3.4 Calling significantly mutated genes

MutSigCV tool was used to identify significantly mutated genes by taking mutations of all samples as input and comparing them against the background mutation rate. Genes mutated more often than expected by chance were regarded as candidates of driver genes. The first 10 most significantly mutated genes and their co-mutation status were analyzed. The software ran with default parameters, and the tool package offered background mutation rate files.

2.3.4.1 Mutational signature analysis

Mutational signature analysis in 6 PTC patients was performed by using the deconstructSigs approach(3) and its R package (deconstructSigs v1.8.0) with default parameters. Thirty COSMIC cancer signatures were considered, contributions (weights) in each patient were normalized between 0 and 1, and signatures below 0.06 were filtered out.

2.4 RNA-Seq

2.4.1 RNA extraction

RNA was extracted from tissues using the TRIzol method (Ambion, Invitrogen, USA) according to the reagent protocols. The concentration and RNA integrity were then determined using a NanoDrop 2000 spectrophotometer (NanoDrop Technologies, Wilmington, USA) and an Agilent 2100 Bioanalyzer (Agilent, CA, USA). RNA samples exhibiting an RNA integrity number (RIN) greater than 6.0 were included in the study. For library preparation of RNA sequencing, a total amount of 1 mg RNA per sample was used as the input material for the RNA sample preparations. Sequencing libraries were generated using NEBNext UltraTM RNA Library Prep Kit for Illumina (#E7530L, NEB, USA) following the manufacturer's recommendations, and index codes were added to attribute sequences to each sample. Briefly, mRNA was purified from total RNA using poly-T oligo-attached magnetic beads. Fragmentation was performed using divalent cations under elevated temperature in NEBNext First Strand Synthesis Reaction Buffer (5X). First-strand cDNA was synthesized using random hexamer primer and RNase H. Second-strand cDNA synthesis was performed using buffer, dNTPs, DNA polymerase I and RNase H. The library fragments were purified with QiaQuick PCR

kits and eluted with EB buffer, followed by terminal repair, A-tailing and adaptor addition. The aimed products were retrieved, PCR was performed, and the library was completed. The RNA concentration of the library was measured using Qubit RNA Assay Kit in Qubit 3.0, then diluted to 1 ng/mL. Insert size was assessed using the Agilent Bioanalyzer 2100 system (Agilent Technologies, CA,USA), and qualified insert size was accurately quantified using StepOnePlusTM Real-Time PCR System (Library valid concentration > 10 nM). The clustering of the index-coded samples was performed on a cBot cluster generation system using HiSeq PE Cluster Kit v4-cBot-HS (Illumina) according to the manufacturer's instructions. After cluster generation, the libraries were sequenced on an Illumina platform, and 150 bp paired-end reads were generated.

2.4.2 RNA-Seq data analysis

RNA-Seq reads were adaptor trimmed, and FastQC (version 0.11.7) software assessed the data quality before any data filtering criteria were applied. Read quantification was performed using Kallisto (v0.45.0), a pseudoalignment-based method to quantify transcript abundance in transcripts per million (TPM) counts, using RefSeq cDNA transcripts in the human reference genome (GRCh38.p12 assembly) for indexing(4). Transcripts with a TPM score above one were retained, resulting in 25,978 gene IDs. All known exons in the annotated file were 100% covered.

2.5 Proteomic analysis

2.5.1 Protein extraction and FASP digestion

Collected samples were supplemented with lysis buffer (2% SDS, 100 mM Tris-HCl, pH 8.0). Samples were boiled at 95 °C for 10 min and then sonicated for 10 min (5 s on and 2 s off). After centrifugation at 20,000 g for 20 min, the supernatants were collected, and the protein concentration was measured using a BCA assay. Extracted proteins were reduced in 100 mM dithiothreitol at 37 °C for 30 min. After reduction, the protein samples were transferred to the Pall Nanosep filter for FASP digestion. After once buffer displacement with 8 M urea and 100 mM Tris-HCl, pH 8.0, the proteins were alkylated with 100 mM iodoacetamide. The subsequent buffer displacement procedure was carried out as follows: three-time with 8 M urea and 100 mM Tris-HCl, twice with 50 mM Tris-HCl in 20% ACN and once with the digestion buffer (50 mM Tris-HCl, pH 8.0). After buffer displacement, the samples were resuspended using digestion buffer and digested using trypsin (enzyme/protein ratio as 1:50 (w/w)) at 37 °C for 12 hours. The peptides were collected by centrifuge, and then the filters were washed twice with 10% ACN. The filtrates were pooled and vacuum-dried by Speed Vac.

2.5.2 Nano-LC-MS/MS analysis

Nano-LC was performed using an EASY-nLC 1200 system (Thermo Fisher Scientific). Samples were loaded and analyzed on an in-house packed C18 columns (75 µm i.d. × ~25 cm; 1.9 µm, ReproSil-Pur 120 C18-AQ, Dr. Maisch GmbH, Germany)(5). The mobile phases consisted of solvent A (0.1% FA) and solvent B (0.1% FA in 80% ACN). The peptides were eluted in the following 120-min gradient: 5-8% B in 3 min, 8-44% B in 100 min, 44-70% B in 5 min, 70-100% B in 2 min, and 100% B for 10 min at a flow rate of 200 nL/min. Mass spectrometry data were collected using an Orbitrap Exploris 480 mass spectrometer with FAIMS Pro device (Thermo Fisher Scientific). Data was acquired using two different CV (-45 and -65 V), and the cycle time was set as 1.5 sec/CV for internal stepping FAIMS experiments. MS1 data were collected with a mass resolution of 120000 (mass range as 350-1600, 300% of AGC target, and auto maximum injection time mode). Precursor

ions with charge states between 2 and 7 were selected for MS2 analysis, and a 60 s dynamic exclusion window was used. MS2 scans were performed with a mass resolution of 7500 (isolation window as 0.7 m/z, NCE as 30%, defined first mass as 110 m/z, and auto maximum injection time mode).

2.5.3 Database searching

The raw data files were analyzed using Proteome Discoverer (version 2.4, Thermo Fisher Scientific) software with the Sequest HT search engine. Human proteome database (202003, 75,004 sequences) was downloaded from UniProt. The processing and consensus workflow was set up according to the incorporated analysis templates. The parameters in the processing workflow were set as: trypsin/P as the enzyme; up to 2 missed cleavage sites were allowed; 10 ppm mass tolerance for MS and 0.05 Da for MS/MS fragment ions; carbamidomethylation on cysteine as fixed modification; oxidation on methionine, oxidation on methionine, acetylation on protein N-Terminal, Met-loss and Met-loss+Acetyl on protein N-Terminal as variable modification. The incorporated Percolator in Proteome Discoverer was used to validate the search results and only the hits with FDR ≤ 0.01 were accepted for further analyses. The 'Minora Feature Detector' node in processing workflow and the corresponded default consensus workflow were applied for label-free analysis.

2.6 Differential genes and protein analysis

In terms of the identification of differentially expressed genes (DEGs), we utilized the edgeR R package to conduct differential analysis, with a defined parameter of |log2(fold change)| > 2 and FDR < 0.001(6). As for differentially expressed proteins (DEPs), we employed the limma R package with |log2(fold change)| > 2 and FDR < 0.05(7). We then collected RNA-seq profiles and clinical information of TCGA-THCA cohorts, and then we matched the samples with clinical data. PTC samples were screened based on primary diagnosis. Heatmaps were drawn through "pheatmap" R package.

2.7 Pathway enrichment analysis

To enrich functional pathways among selected DEGs and DEPs, clusterProfiler R package was used to conduct Gene Ontology (GO) and Kyoto Encyclopedia of Genes and Genomes (KEGG) analysis, and a q-value < 0.05 was set as the cutoff standard(8). The enrichplot R package was responsible for the visualization.

2.8 Weighted gene co-expression network analysis(WGCNA)

The WGCNA R package was applied to the genes (n=4,828) with FDR<0.05 in differential analysis9 to identify differentially co-expressed gene modules.(9). The eigengenes of each module were used to measure the association between a module and clinical information. To further investigate, we defined the gene significance (GS) as the correlation between gene expression profiles and the eigengene of each module. Additionally, the module membership (MM) was defined as the absolute value of the correlation between genes and the phenotypic trait, in this case, tumor size. We exported the edge data of genes within significant modules for further analysis and visualization.

2.9 Protein-protein interactions (PPI) network and screening for hub genes

Based on identified DEPs, we construct a PPI network through the Search Tool for the Retrieval of Interacting Genes Database (STRING)(https://cn.string-db.org/)(10). The edge data from WGCNA and PPI network were visualized through Cytoscape (version 3.10.0)(11). We first removed those genes outside the main network and used cytoHubba, a plugin in Cytoscape, to screen for hub genes. Nodes were ranked based on maximal clique centrality (MCC), and the top8 nodes were selected as candidate hub genes from the WGCNA and PPI networks, respectively. Venn diagram R package was used to depict the overlap among somatic alterations, DEGs, and DEPs and the overlap genes of those three profiles were regarded as hub genes(12).

2.10 Prognostic verification of hub genes

Before validating the prognostic potential of hub genes, we first examined the expression level between thyroid cancers and normal tissue derived from the TCGA and GTEx database through TNMplot (https://tnmplot.com/analysis/)(13). Then, the Kaplan-Meier plotter (http://www.kmplot.com/) was employed for survival analysis based on TCGA-THCA (n=502). The best cutoff was automatically determined through this tool. The observed outcomes were overall survival (OS) and recurrence-free survival (RFS).

2.11 ROC analysis and logistic regression model

We conducted receiver operating characteristic (ROC) analysis of hub genes with 475 PTC samples and 59 normal samples from TCGA-THCA through pROC R pacakge(14). According to the events per variable (EPV) rule, the number of variables in the model should be less than 1/10 of the observed events frequency, indicates we ought to select less than 6 variables. Thus, hub genes with an area under the curve (AUC) > 0.85 were qualified to construct a logistic regression model. The glmnet package with the family="binomial" parameter was used for logistic regression analysis, and stepwise regression was performed using the step function(15). The forest plot was obtained through the forest plot R package. A nomogram was produced through rms R package(16). We tested the model with GSE33630 (49 PTC and 45 normal samples) datasets. The pan-cancer expression level of those genes composed of the model was shown throughTNMplot(13). GSE63514 (28 cervical squamous cell carcinoma samples and 100 non-tumor samples), GSE132305 (182 extrahepatic cholangiocarcinomas and 38 non-tumoral bile duct samples), GSE53757 (72 clear cell renal tumor samples and 72 normal tissue samples), GSE121248 (70 hepatocellular carcinoma samples and 37 adjacent normal tissues), GSE43458 (80 lung adenocarcinomas and 30 normal lung tissues) datasets were used to verify the specificity for PTC of this model. In GSE63514, normal tissues, cervical intraepithelial neoplasia (CIN) I, CIN II, CIN III and were considered non-tumor samples.

3 The raw data repository

The raw sequence data reported in this paper have been deposited in the Genome Sequence Archivein National Genomics Data Center, China National Center for Bioinformation / Beijing Institute of Genomics, Chinese Academy of Sciences (GSA-Human: HRA005389) that are publicly accessible at https://ngdc.cncb.ac.cn/gsa-human(17, 18).

The mass spectrometry proteomics data have been deposited to the ProteomeXchange Consortium (http://proteomecentral.proteomexchange.org) via the iProX partner repository with the dataset identifier PXD044815(19, 20).

4 Abbreviated terms

A1BG: Alpha-1B-glycoprotein. ABR: Active breakpoint cluster region-related protein. AEBP1: Adipocyte enhancer-binding protein 1. AHNAK2: AHNAK nucleoprotein 2. AML: Acute myelocytic leukemia. ANK3: Ankyrin-3. AUC: Area under the curve. BRAF: Serine/threonine-protein kinase B-raf. BRCA: Breast cancer.C2CD2L: Phospholipid transfer protein C2CD2L. CD34: Hematopoietic progenitor cell antigen CD34. CDC42EP5: Cdc42 effector protein 5. CESC: Cervical cancer. CHEK2: Serine/threonine-protein kinase Chk2. CHOL: Bile duct cancer. CI: confidence interval. COL12A1: Collagen alpha-1(XII) chain. CTSA: Lysosomal protective protein. DDA: Data-dependent acquisition. DEGs: Differentially expressed genes. DEPs: Differentially expressed proteins. EBLN2: Endogenous bornavirus-like nucleoprotein 2. ECM: Extracellular matrix. ESCA: Esophageal cancer. EVP: Events per variable. FDR: False discovery rate. FFPE: Formalin fixed paraffin embedded. FVPTC: Follicular variants of papillary thyroid carcinoma. GATK: Genome Analysis Toolkit. GEO: NCBI Gene expression omnibus. GO: Gene Ontology. GPX1: Glutathione peroxidase 1. GYPA: Glycophorin-A. ITGA2B: Calcium and integrin-binding protein 1. LASP1: LIM and SH3 domain protein 1. LIHC: Liver hepatocellular carcinoma. LUAD/Lung_AC: Lung adenocarcinoma. Lung_SC: Lung squamous cell carcinoma. KEGG: Kyoto Encyclopedia of Genes and Genomes. KIRC: Kidney renal clear cell carcinoma. KRT19: Keratin, type I cytoskeletal 19. KRTAP5-7: Keratin-associated protein 5-7. MAGEB16: Melanoma-associated antigen B16. MAPK: Mitogen-activated protein kinase. NF1: Neurofibromin. NPAP1: Nuclear pore-associated protein 1. NrCAM: Neuronal cell adhesion molecule. OR51M1: Olfactory receptor 51M1. OR: Odds ratio. OS: Overall survival. PCA: Principal component analysis. PCSK9: Proprotein convertase subtilisin/kexin type 9. PMS2: Mismatch repair endonuclease PMS2. PNPLA5: Patatin-like phospholipase domain-containing protein 5. POSTN: Periostin. PPI: protein-protein interaction. PTC: Papillary thyroid carcinoma. Renal_CC: Renal clear cell carcinoma. Renal_CH: Renal chromo phobe cell carcinoma. Renal_PA: Renal papillary cell carcinoma. RFS: Recurrence-free survival. RNA-seq: RNA sequencing. RAS: Rat sarcoma. ROC: Receiver operating characteristic curve. RPKM: Transcript per million mapped. RTK: Receptor tyrosine kinase. scRNA-seq: Single-cell RNA sequencing. SFN: Stratifin. SGIP1: SH3-containing GRB2-like protein 3-interacting protein 1. SNV: Single nucleotide variant. TC: Thyroid cancer. TCGA: The cancer genome atlas. THBS2: Thrombospondin-2. THCA: Thyroid carcinoma. TMB: Tumor mutation burden. TPO: Thyroid peroxidase. Uterus_CS: Uterine Carcinosarcoma. Uterus_EC: Uterine Corpus Endometrial Carcinoma. WES: Whole-exome sequencing. WGCNA: Weighted gene co-expression network analysis. ZNF714: Zinc finger protein 714.

5 Reference

1. Li, H. and Durbin, R. Fast and accurate short read alignment with Burrows-Wheeler transform. *Bioinformatics.* 2009 **25** 1754-60. https://doi.org/10.1093/bioinformatics/btp324.
2. Li, H., Handsaker, B., Wysoker, A., Fennell, T., Ruan, J., Homer, N., Marth, G., Abecasis, G., Durbin, R., and Genome Project Data Processing, S. The Sequence Alignment/Map format and SAMtools. *Bioinformatics.* 2009 **25** 2078-9. https://doi.org/10.1093/bioinformatics/btp352.
3. Xu, J.Y., Zhang, C., Wang, X., Zhai, L., Ma, Y., Mao, Y., Qian, K., Sun, C., Liu, Z., Jiang, S., Wang, M., Feng, L., Zhao, L., Liu, P., Wang, B., Zhao, X., Xie, H., Yang, X., Zhao, L., Chang, Y., Jia, J., Wang, X., Zhang, Y., Wang, Y., Yang, Y., Wu, Z., Yang, L., Liu, B., Zhao, T., Ren, S., Sun, A., Zhao, Y., Ying, W., Wang, F., Wang, G., Zhang, Y., Cheng, S., Qin, J., Qian, X., Wang, Y., Li, J., He, F., Xiao, T., and Tan, M. Integrative Proteomic Characterization of Human Lung Adenocarcinoma. *Cell.* 2020 **182** 245-261 e17. https://doi.org/10.1016/j.cell.2020.05.043.
4. Pertea, M., Kim, D., Pertea, G.M., Leek, J.T., and Salzberg, S.L. Transcript-level expression analysis of RNA-seq experiments with HISAT, StringTie and Ballgown. *Nat Protoc.* 2016 **11** 1650-67. https://doi.org/10.1038/nprot.2016.095.
5. Kovalchuk, S.I., Jensen, O.N., and Rogowska-Wrzesinska, A. FlashPack: Fast and Simple Preparation of Ultrahigh-performance Capillary Columns for LC-MS. *Mol Cell Proteomics.* 2019 **18** 383-390. https://doi.org/10.1074/mcp.TIR118.000953.
6. Robinson, M.D., Mccarthy, D.J., and Smyth, G.K. edgeR: a Bioconductor package for differential expression analysis of digital gene expression data. *Bioinformatics.* 2010 **26** 139-40. https://doi.org/10.1093/bioinformatics/btp616.
7. Ritchie, M.E., Phipson, B., Wu, D., Hu, Y., Law, C.W., Shi, W., and Smyth, G.K. limma powers differential expression analyses for RNA-sequencing and microarray studies. *Nucleic Acids Res.* 2015 **43** e47. https://doi.org/10.1093/nar/gkv007.
8. Wu, T., Hu, E., Xu, S., Chen, M., Guo, P., Dai, Z., Feng, T., Zhou, L., Tang, W., Zhan, L., Fu, X., Liu, S., Bo, X., and Yu, G. clusterProfiler 4.0: A universal enrichment tool for interpreting omics data. *Innovation (Camb).* 2021 **2** 100141. https://doi.org/10.1016/j.xinn.2021.100141.
9. Langfelder, P. and Horvath, S. WGCNA: an R package for weighted correlation network analysis. *BMC Bioinformatics.* 2008 **9** 559. https://doi.org/10.1186/1471-2105-9-559.
10. Szklarczyk, D., Gable, A.L., Nastou, K.C., Lyon, D., Kirsch, R., Pyysalo, S., Doncheva, N.T., Legeay, M., Fang, T., Bork, P., Jensen, L.J., and Von Mering, C. The STRING database in 2021: customizable protein-protein networks, and functional characterization of user-uploaded gene/measurement sets. *Nucleic Acids Res.* 2021 **49** D605-d612. https://doi.org/10.1093/nar/gkaa1074.
11. Shannon, P., Markiel, A., Ozier, O., Baliga, N.S., Wang, J.T., Ramage, D., Amin, N., Schwikowski, B., and Ideker, T. Cytoscape: a software environment for integrated models of biomolecular interaction networks. *Genome Res.* 2003 **13** 2498-504. https://doi.org/10.1101/gr.1239303.
12. Chen, H. and Boutros, P.C. VennDiagram: a package for the generation of highly-customizable Venn and Euler diagrams in R. *BMC Bioinformatics.* 2011 **12** 35. https://doi.org/10.1186/1471-2105-12-35.
13. Bartha, A. and Gyorffy, B. TNMplot.com: A Web Tool for the Comparison of Gene Expression in Normal, Tumor and Metastatic Tissues. *Int J Mol Sci.* 2021 **22**. https://doi.org/10.3390/ijms22052622.
14. Robin, X., Turck, N., Hainard, A., Tiberti, N., Lisacek, F., Sanchez, J.C., and Müller, M. pROC: an open-source package for R and S+ to analyze and compare ROC curves. *BMC Bioinformatics.* 2011 **12** 77. https://doi.org/10.1186/1471-2105-12-77.
15. Friedman, J., Hastie, T., and Tibshirani, R. Regularization Paths for Generalized Linear Models via Coordinate Descent. *J Stat Softw.* 2010 **33** 1-22.
16. Liu, Y., Li, L., Jiang, D., Yang, M., Gao, X., Lv, K., Xu, W., Wei, H., Wan, W., and Xiao, J. A Novel Nomogram for Survival Prediction of Patients with Spinal Metastasis From Prostate Cancer. *Spine (Phila Pa 1976).* 2021

46 E364-e373. https://doi.org/10.1097/brs.0000000000003888.

17. Members, C.-N. and Partners. Database Resources of the National Genomics Data Center, China National Center for Bioinformation in 2022. *Nucleic Acids Res.* 2022 **50** D27-D38. https://doi.org/10.1093/nar/gkab951.
18. Chen, T., Chen, X., Zhang, S., Zhu, J., Tang, B., Wang, A., Dong, L., Zhang, Z., Yu, C., Sun, Y., Chi, L., Chen, H., Zhai, S., Sun, Y., Lan, L., Zhang, X., Xiao, J., Bao, Y., Wang, Y., Zhang, Z., and Zhao, W. The Genome Sequence Archive Family: Toward Explosive Data Growth and Diverse Data Types. *Genomics Proteomics Bioinformatics.* 2021 **19** 578-583. https://doi.org/10.1016/j.gpb.2021.08.001.
19. Chen, T., Ma, J., Liu, Y., Chen, Z., Xiao, N., Lu, Y., Fu, Y., Yang, C., Li, M., Wu, S., Wang, X., Li, D., He, F., Hermjakob, H., and Zhu, Y. iProX in 2021: connecting proteomics data sharing with big data. *Nucleic Acids Res.* 2022 **50** D1522-D1527. https://doi.org/10.1093/nar/gkab1081.
20. Ma, J., Chen, T., Wu, S., Yang, C., Bai, M., Shu, K., Li, K., Zhang, G., Jin, Z., He, F., Hermjakob, H., and Zhu, Y. iProX: an integrated proteome resource. *Nucleic Acids Res.* 2019 **47** D1211-D1217. https://doi.org/10.1093/nar/gky869.

6 Supplementary Figures and Tables

6.1 Supplementary Figures

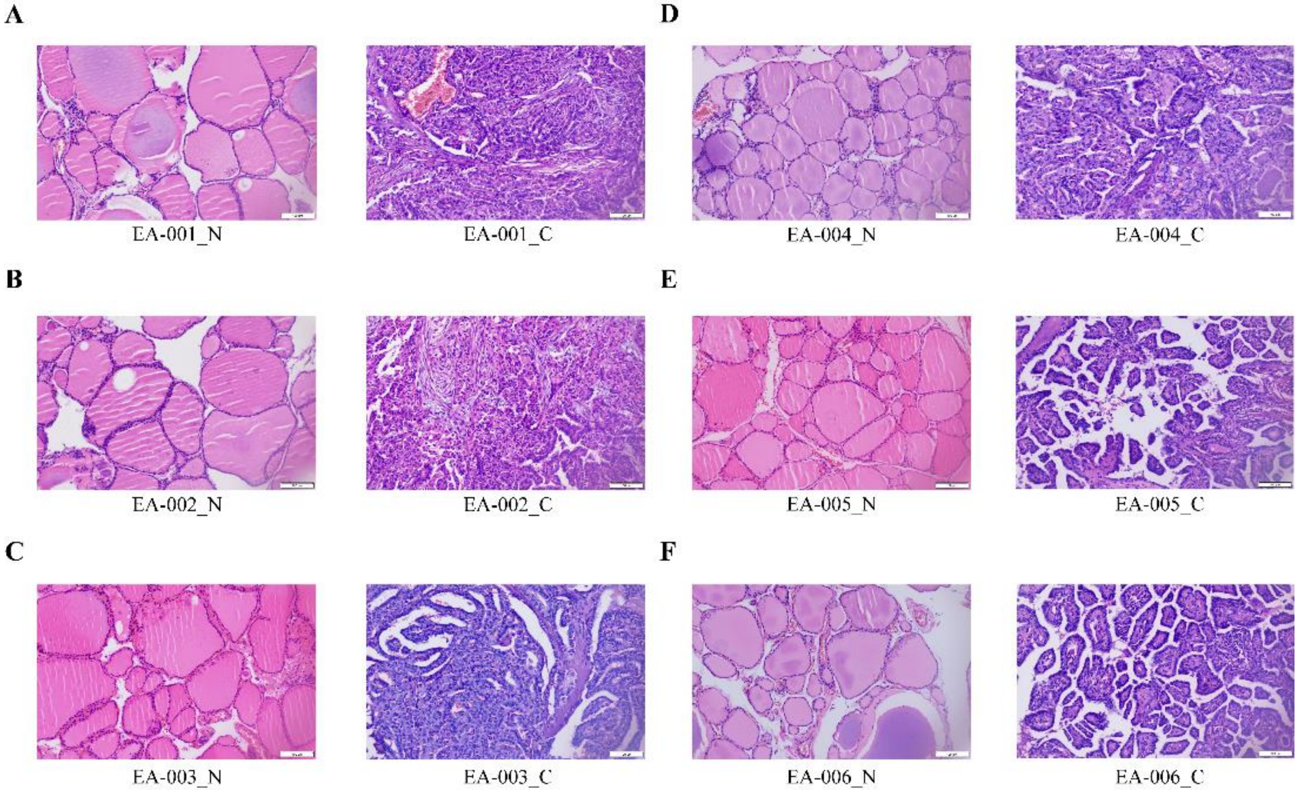

Supplementary Figure S1 Paired freezing microtome section specimens from 6 patiens were HE-stained. (A). EA-001_N/C. (B). EA-002_N/C. (C). EA-003_N/C. (D). EA-004_N/C. (E). EA-005_N/C. (F). EA-006_N/C. N is paired peritumor tissues, C is the primary tumor tissues.

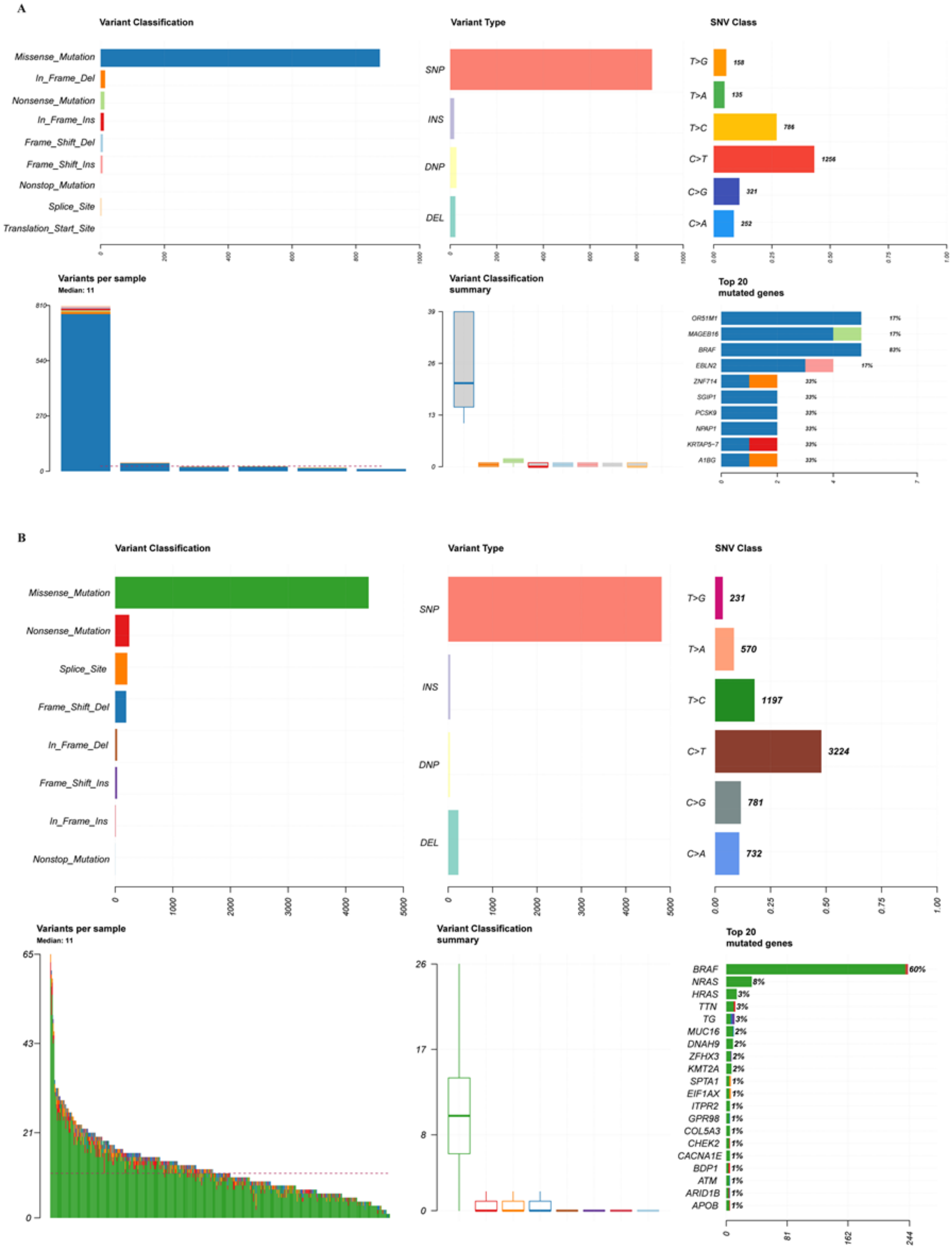

Supplementary Figure S2 Summary of somatic alterations in local patients and TCGA-THCA cohorts. (A). in local patients, and (B). in TCGA-THCA cohorts. The variant classification, variant types, SNV class, variant per sample, variant classification summary, and top 20 mutated genes in local patients and TCGA-THCA cohorts.

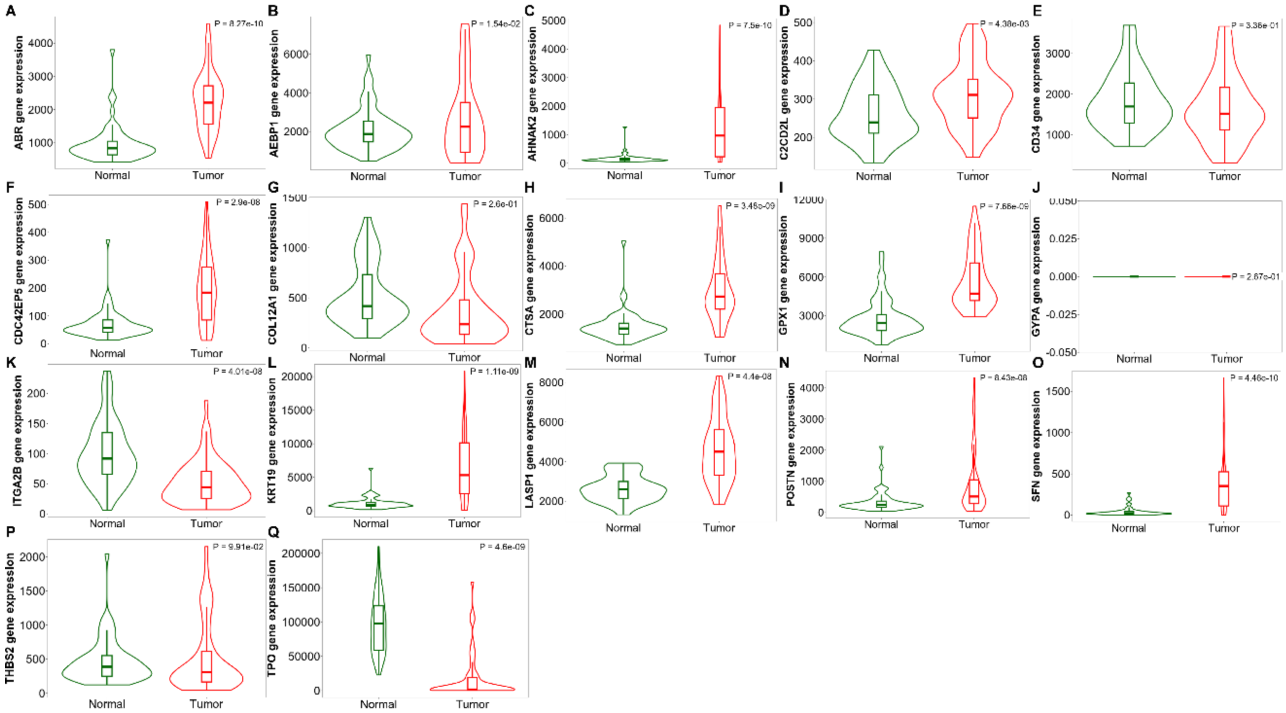

Supplementary Figure S3 Expression levels of 17 hub genes between normal tissue and PTC. Data are based on TCGA-THCA, drawn by TNMplot. (A). *ABR* (upregulated). (B). *AEBP1*. (C). *AHNAK2* (upregulated). (D). *C2CD2L*. (E). *CD34*. (F). *CDC42EP5* (upregulated). (G). *COL12A1*. (H). *CTSA* (upregulated). (I). *GPX* 1(upregulated). (J). *GYPA*. (K). *ITGA2B* (downregulated). (L). *KRT19* (upregulated). (M). *LASP1* (upregulated). (N). *POSTN* (upregulated). (O). *SFN* (upregulated). (P). THBS2, and (Q). *TPO* (downregulated).The genes with significant differences are highlighted with upregulated or downregulated.

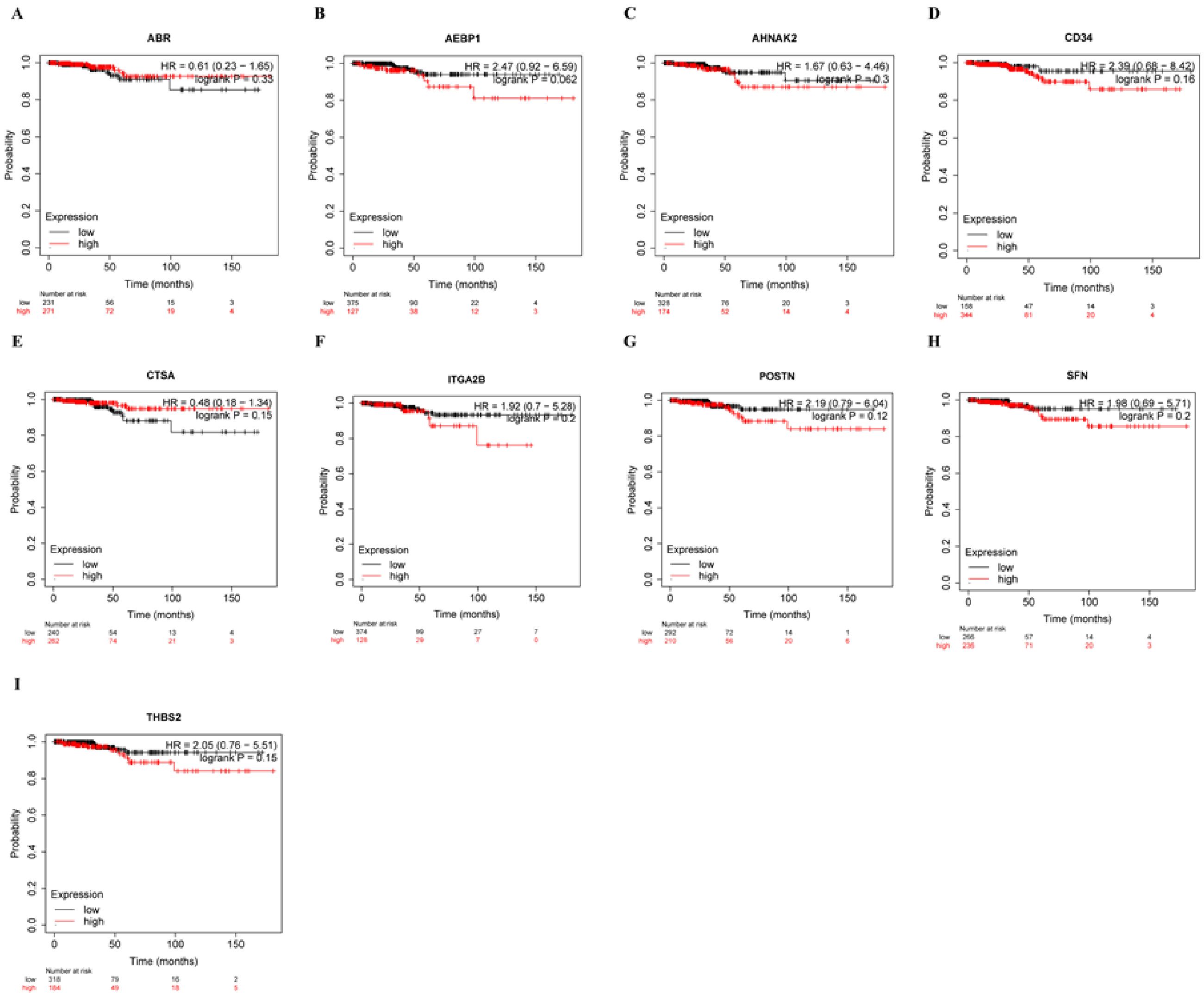

Supplementary Figure S4 Survival analysis on K-M plotter based on 502 patients from TCGA-THCA cohorts revealed associations between the expression of hub genes and OS. (A). *ABR*. (B). *AEBP1*. (C). *AHAK2*. (D) *CD34*. (E). *CTSA*. (F). *ITGA2B*. (G). *POSTN*. (H). *SFN*. (I). *THBS2*. Insignificant hub genes were shown here.

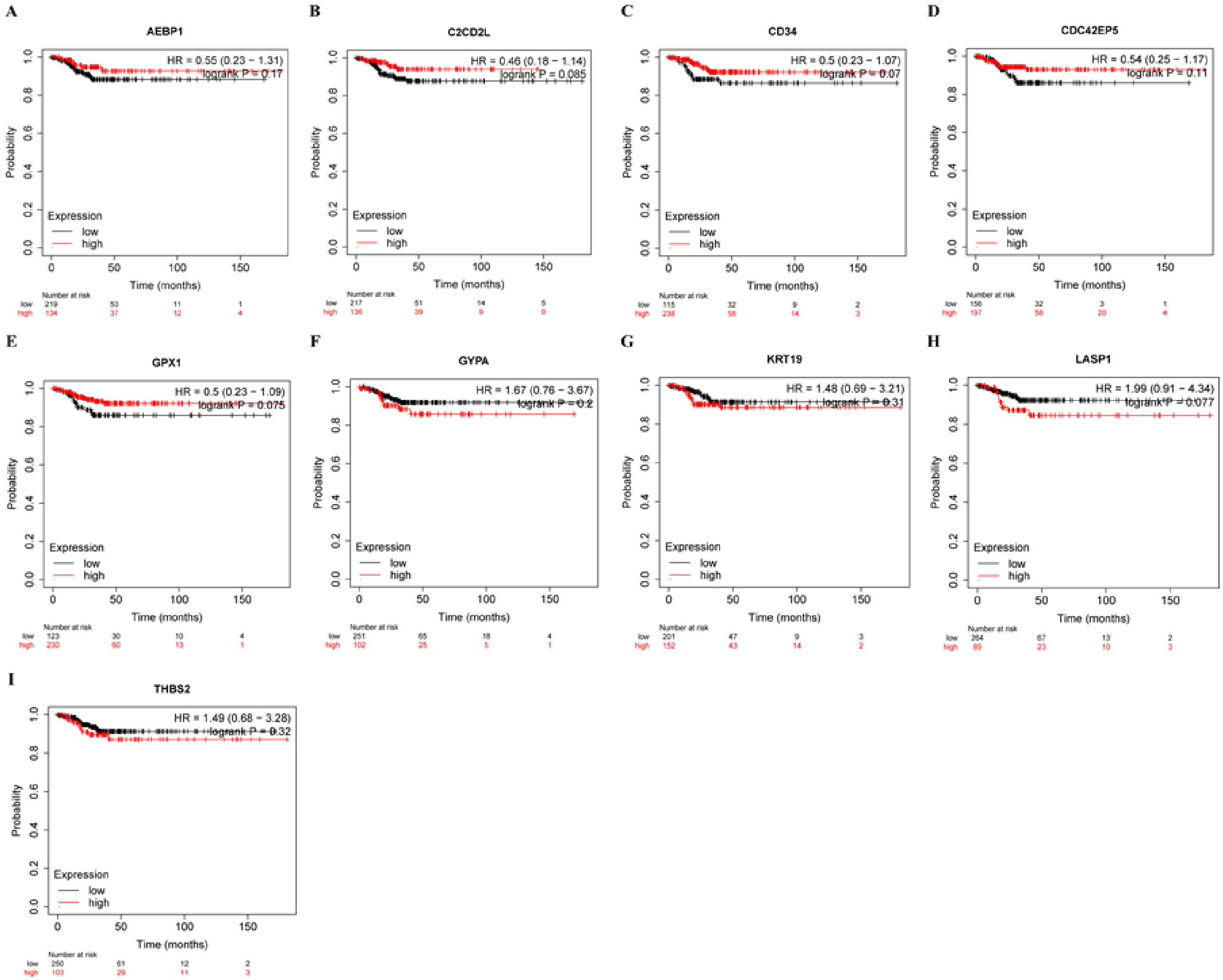

Supplementary Figure S5 Survival analysis on K-M plotter based on 502 patients from TCGA-THCA cohorts revealed associations between the expression of hub genes and RFS. (A). *AEBP1*. (B). *C2CD2L*. (C). *CD34*. (D). *CDC42EP5*. (E). *GPX1*. (F). *GYPA*. (G). *KRT19*. (H). *LASP1*. (I). *THBS2*. Insignificant hub genes were shown here.

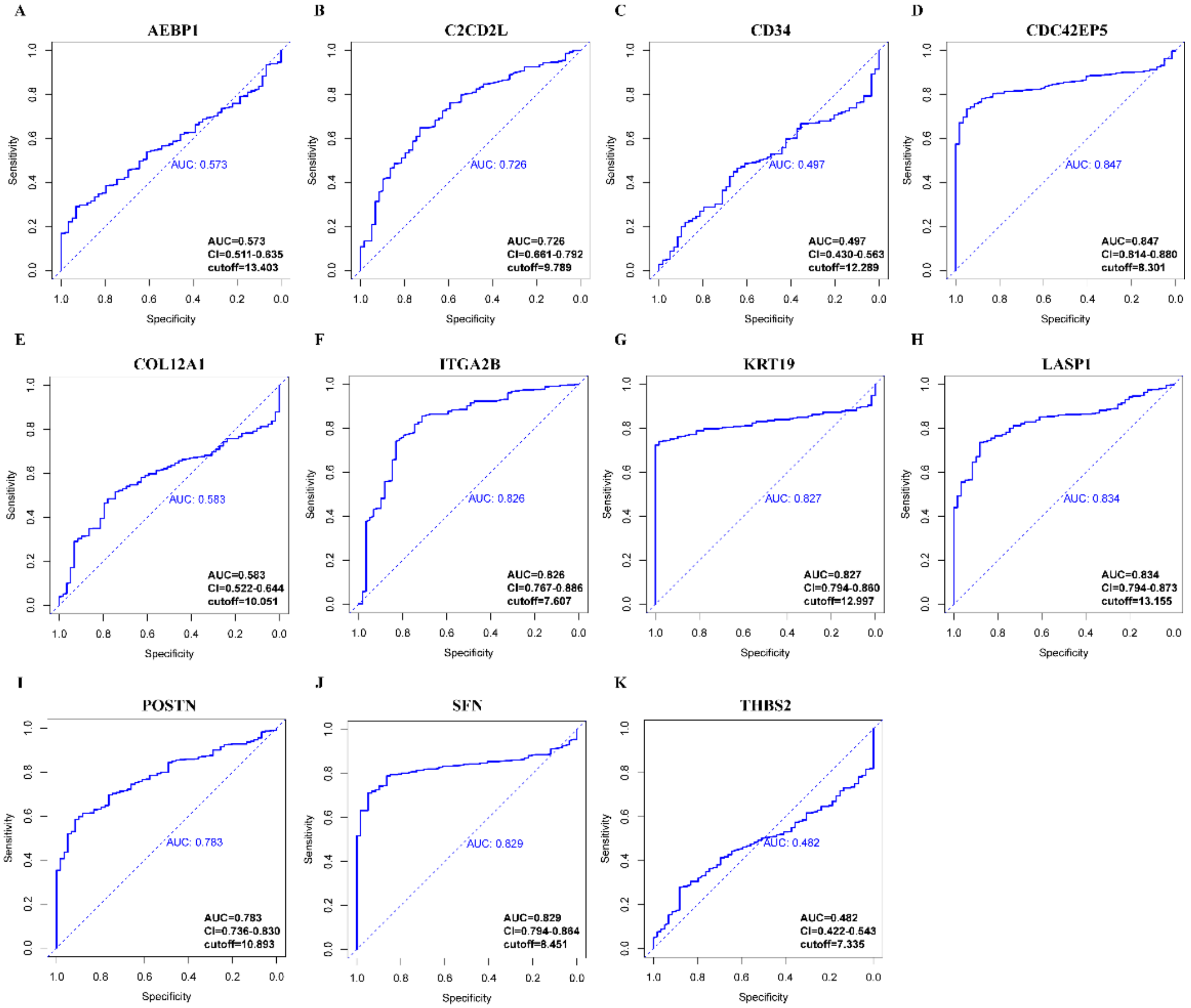

Supplementary Figure S6 ROC analysis of hub genes with AUC value < 0.85. (A). *AEBP1*. (B). *C2CD2L*. (C). *CD34*. (D). *CDC42EP5*. (E). *COL12A1*. (F). *ITGA2B*. (G). *KRT19*. (H). *LASP1*. (I). *POSTN*. (J). *SFN*. (I). *THBS2*. Insignificant hub genes were shown here.

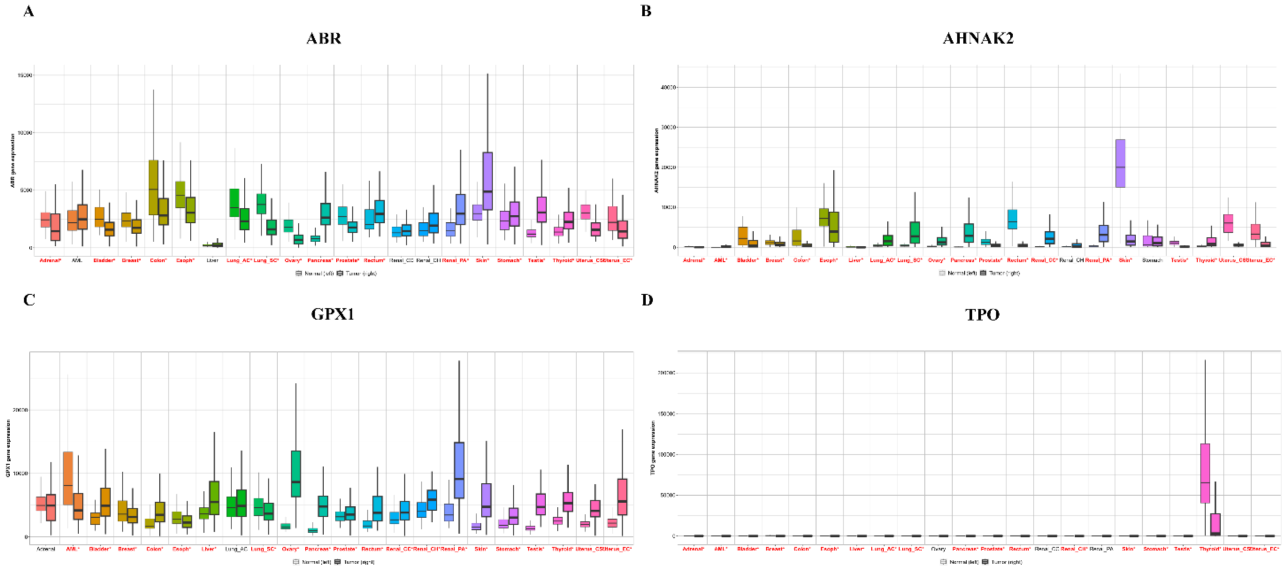

Supplementary Figure S7 Pan-cancer expression levels of four genes composed of predictive model derived from TNMplot. Significant differences by Mann-Whitney U test are marked with red*. (**A**). *ABR*. (**B**). *AHNAK2*. (**C**). *GPX1*. (**D**). *TPO*.

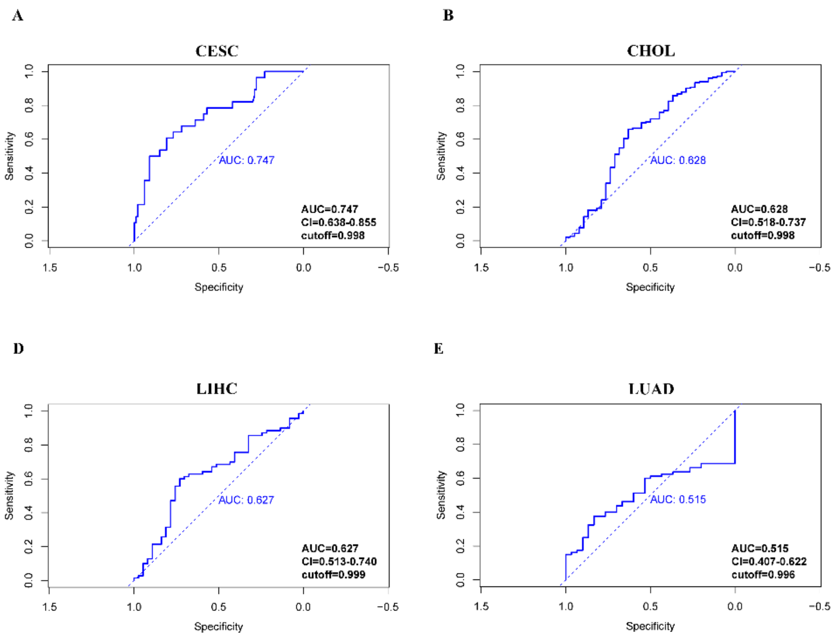

Supplementary Figure S8 ROC analysis of predictive model applied on other types of cancer. (**A**). CESC (cervical squamous cell carcinoma). (**B**). CHOL (cholangiocarcinoma tumor). (**C**). KIRC (kidney renal clear cell carcinoma). (**D**). LIHC (liver hepatocellular carcinoma). (**E**). LUAD (lung adenocarcinoma).

6.2 Supplementary Tables

Supplementary Table 1: The clinical baseline information,

Supplementary Table 2: The RNA-Seq raw data,

Supplementary Table S: The proteins raw data.
